# Improving Behavioral Healthcare Access Disparities by Training Providers in Disadvantaged Communities — Evidence of Strategy Effectiveness

**DOI:** 10.1007/s11606-024-09020-1

**Published:** 2024-10-31

**Authors:** Ariel B. Neikrug, Shreya S. Cho, Ethan S. Nguyen, Annamarie Stehli, Shutong Huo, Careesa Garcia, Stephanie Au, Mandana Masoumirad, Wendy Cant, Khanh-Van Le-Bucklin, Jane P. Gagliardi, Glen L. Xiong, Robert M. McCarron

**Affiliations:** 1 Department of Psychiatry and Human Behavior, University of California Irvine, Irvine, CA USA; 2 Program in Public Health, University of California Irvine, Irvine, CA USA; 3 Office of Medical Education, School of Medicine, University of California Irvine, Irvine, CA USA; 4 University of California, Irvine, Sue & Bill Gross School of Nursing, Irvine, CA USA; 5 Psychiatry and Behavioral Sciences, Duke University School of Medicine, Durham, NC USA; 6 Department of Psychiatry and Behavioral Sciences, University of California Davis, Sacramento, CA USA

**Keywords:** healthcare disparity, behavioral health, mental health, disadvantage, health professional shortage areas

## Abstract

**Background:**

Inadequate access to behavioral health services disproportionately impacts marginalized populations who live in disadvantaged areas. To reduce this gap, programs dedicated to optimizing behavioral health education and training must focus their efforts to enroll providers who practice in these disadvantaged areas.

**Objective:**

The Train New Trainers (TNT) fellowship program aims to enhance behavioral health knowledge, skills, and attitudes of primary care providers (PCPs) who deliver care in disadvantaged communities. We evaluate the effectiveness of the TNT recruitment strategy and the use of scholarships for targeting and recruiting PCPs who practice in disadvantaged communities.

**Design:**

Observational study.

**Participants:**

TNT fellows from 2016 to 2023.

**Main Measures:**

State/federal classifications of medically underserved counties were used to establish scholarship criteria. Area Deprivation Index (ADI) was utilized to provide criterion validity for the use of state/federal criteria in the recruitment strategy, and to evaluate the effectiveness of the program in successfully recruiting PCPs practicing in disadvantaged communities.

**Key Results:**

Practice location data were available for 347 fellows, 88.8% of whom received scholarships. Of the 347 practices, 300 (86.5%) primarily served communities meeting at least one state or federal criterion for medical shortage areas and/or underserved areas. According to ADI scores, 32.3% of practices served areas classified in the highest ADI (ADI decile 9 or 10), with a progressive increase in the proportion of fellows practicing in underserved areas each year; in 2023, 89.9% of practices met federal shortage criteria and 40.5% served areas with the highest deciles of ADI.

**Conclusions:**

The TNT program strategy for recruiting PCPs from high medical need geographical areas is associated with bringing primary care psychiatry education to areas considered underserved and disadvantaged. Equipping PCPs practicing in underserved areas with enhanced knowledge and skills in behavioral medicine has the potential to significantly improve the existing access gap in disadvantaged communities.

## INTRODUCTION

There is a growing demand for access to mental and behavioral health (hereon referred to as behavioral health) services in the United States (US).^1,2^ In 2021, approximately 23% of adults aged 18 and older reported experiencing a mental illness within the past year, marking an increase from 18% in 2015.^3,4^ Yet, only 47.2% of individuals dealing with mental illness were able to access behavioral health services.^3^ Access to healthcare is particularly a concern in California with 52 out of 58 State counties having areas that meet federal criteria for Health Professional Shortage Areas (HPSA), which can be designated by facility or reflect a specific geographic areas, not necessarily entire counties.^5^ Nonetheless, more than 13 million Californians live in an area with an inadequate supply of behavioral health professionals,^6^ where locating a behavioral health specialist is twice as difficult as finding a PCP.^7^

As the need for behavioral health services increases and the access to specialty behavioral healthcare continues to decline, PCPs are increasingly assigned the responsibility of delivering behavioral healthcare services to their patients.^8,9^ It is estimated that PCPs treat nearly 75% of behavioral healthcare patients and are responsible for more than 75% of antidepressant prescriptions.^10–12^ In 2019, California PCPs were involved in 73% of behavioral healthcare received by adults with serious psychological distress, a 6% increase since 2015.^13^ The demand for PCPs to deliver behavioral healthcare is anticipated to rise further due to the substantial impact of COVID-19 on overall health and the significant increase in mental illness during and after the pandemic.^14,15^

While existing approaches for improving access to behavioral health services (e.g., coordinated care, co-located care, and integrated care) have been helpful,^16^ they are dependent on the availability of behavioral health specialists (i.e., psychiatrists, psychologists, social workers) to consult directly or indirectly on patient care.^16–18^ Such strategies may be particularly impractical in underserved areas given the lack of specialty services.^19,20^ To enhance access to quality behavioral healthcare, there is a need to concentrate behavioral health training efforts on PCPs practicing in disadvantaged and underserved areas. Currently, practical strategies for recruiting PCPs from these underserved areas to participate in training programs are not widely reported or empirically evaluated. This study aims to assess the effectiveness of a systematic recruitment strategy designed to identify and engage PCPs practicing in socioeconomically disadvantaged areas and enroll these practitioners in a comprehensive fellowship program specifically tailored for behavioral healthcare training. This evaluation contributes valuable insights into strategies for enhancing the participation of PCPs from underserved regions in such training initiatives and to reduce behavioral healthcare access disparities.

## METHODS

The University of California, Irvine, Train New Trainers (TNT) primary care psychiatry fellowship addresses the access disparity in behavioral healthcare by strategically focusing its outreach on PCPs serving patients in disadvantaged areas. Started in 2016, this 1-year, multi-faceted program was shown to improve fellows’ knowledge in general psychiatry as well as improve attitudes towards provision of behavioral healthcare services.^12^ The program is geared towards medical providers in primary care settings, such as and including those working in internal medicine, family medicine, emergency medicine, pediatrics, obstetrics and gynecology, neurology, or pharmacy. Fellows must be licensed physicians (MD or DO), nurse practitioners (NP), or physician associates (PA). TNT has a growing expert panel of boarded providers and in 2024 included 25 teaching faculty (20 have a dual board certification in psychiatry and internal/family medicine) who practice in various institutions across the US. The TNT fellowship utilizes published materials and includes over 60 h of specialized training through in-person conferences, live virtual webinars, group mentoring, and open office hours. Participation in mandatory sessions is required for certification, with provisions for making up missed sessions. The program design allows for flexibility in structure (e.g., number of mentorship groups or conference breakout sessions), which supports a robust education that prepares trainees to manage psychiatric and behavioral conditions in primary care. Graduates receive a certificate from the UCI School of Medicine and benefit from lifelong learning opportunities. Participation in this fellowship is possible by either receiving TNT-specific scholarships or paying full tuition. Given the flexible and high-capacity structure of the program (e.g., small group sessions, online content, large faculty body), no capacity limits were set in advance, allowing participation for all eligible applicants.

### TNT Recruitment Strategy

The recruitment strategy for the TNT fellowship program is multi-component and strategically designed to attract PCPs from underserved and disadvantaged areas, while still maintaining a broad marketing appeal. Global marketing efforts included participation in regional and national conferences and exhibitions, such as those hosted by the American Psychiatric Association and American College of Physicians. Additionally, the program leveraged social media platforms like Doximity and LinkedIn to increase brand visibility and engage with PCPs across the country.

Alongside this global marketing effort, local ties were developed with community stakeholders. This entailed fostering relationships at the regional, statewide, and national levels to support recruitment efforts and invest in improving behavioral healthcare access. Beginning in California, the program initiated partnerships with local and state health entities, such as Inland Empire Health Plan, Alameda County, and LA Care, aiming to identify and train PCPs within their networks. Over time, TNT expanded its reach beyond California, establishing contracts with stakeholders in other states (e.g., Kentucky) and even internationally (e.g., United Arab Emirates).

Importantly, the recruitment strategy included a targeted approach to reach even more disadvantaged areas. This involved developing specific initiatives and partnerships tailored to address the unique needs of underserved communities. The primary strategy to attract and retain PCPs from such areas was to provide partial or complete program scholarships to those practicing in underserved counties. By strategically partnering with organizations, the program was able to offer scholarships to PCPs within the areas governed by the partners, with criteria set in collaboration based on federal and state determinations for shortage areas. The stakeholders fund the fellows directly after sponsor verification and pay the TNT program per approved scholarship. Program awards and scholarships are negotiated with stakeholders but administered by the sponsor (see Appendix Table 4 for details).

The work locations of the fellows were abstracted from their application forms. This reported address was used by TNT staff to assess whether the location fell within a pre-defined shortage areas and met the criteria for scholarships. If the criteria were met, this information was transferred to the sponsor, along with the fellow’s provided referrals and contact information for a person who could verify their employment at the specified location. The verification of the work location was conducted solely by the sponsor. Additionally, there could be many reasons for being denied a fellowship, including the requirement by some sponsors to maintain a specific patient load within shortage areas or specific program affiliations. TNT was informed only about those who were approved and did not receive details of the verification outcomes.

Going beyond the 12-month program, TNT fosters continued relationships with fellows even after the end of the fellowship. Graduates are encouraged to attend open seminars, webinars, and take advantage of mentorship office hours provided by the TNT program to both current and past trainees. As a result, TNT alumni have proven to be valuable partners in recruitment efforts, informing other providers within their networks or associations of the TNT fellowships, thus increasing awareness and interest in TNT among new providers.

For this study, TNT’s fellows’ practice location addresses and scholarship information were extracted from program applications for all cohorts 2016–2023. Primary practice location addresses were cross-referenced and validated by the research team using mapping software (e.g., Google Maps, business listings) to ensure the address belonged to a healthcare facility. Only California addresses were included in the analysis, and addresses were excluded if they were residential, associated with a P.O. box, or were not able to be validated. Validated work addresses were used to obtain census tract ID numbers using the HPSA-search tool from the California Department of Health Care Access and Information (HCAI) website^21^ and were subsequently linked to publicly available healthcare databases and outcome metrics of advantage/disadvantage as described below. This study was reviewed and approved by the University of California, Irvine Institutional Review Board.

#### Measures of Advantage/Disadvantage and Underserved Communities

Health Resources & Services Administration (HRSA)^22^ metrics were used to establish the scholarship eligibility criteria. Three types of federal designations were used: Health Professional Shortage Areas (HPSAs), Federally Qualified Health Center (FQHC), and Medically Underserved Areas (MUAs).

#### Health Professional Shortage Areas (HPSAs)

HPSAs are defined as a population, area, or facility with a scarcity of healthcare professionals and services.^23^ HPSA designations are based on federally defined criteria.^22^ Generally, status as a shortage area is based on several factors indicating availability of providers/services relative to need (e.g., population-to-provider ratio, percent of population below 100% of the Federal Poverty Level, and travel time to the nearest source of care outside the HPSA designation area).^22^ Here, three different subtypes of HPSA status were used based on healthcare specialty: Health Professional Shortage Area designated for Primary Care (HPSA PC), Health Professional Shortage Area designated for Mental Health, (HPSA MH), and Primary Care Shortage Area (PCSA).^22,24^ The HPSA MH criteria are specific to professionals classified as mental health providers, and HPSA PC classification includes unique criteria related to providers in defined settings (therefore does not completely overlap with PCSA designations).^24^ Thus, each primary practice location could meet criteria on any combination of the three subtypes or none.

Federal designations are recognized and utilized by the states. Within California, HPSA data are managed and made publicly available by HCAI.^25^ Specifically, HCAI maintains datasets containing socio-demographic and health service information including indicators of a region’s status on each of the three HPSA designations. These indicators were mapped to our TNT primary practice locations via census tract ID numbers.

#### Federally Qualified Health Center (FQHC) and Population-Level Designations

Healthcare facilities can have a designation of being a FQHC separate from its geographic location.^22^ This is also true of facilities serving specific communities (e.g., migrant farm workers). Status as a shortage area is considered automatic in these cases. A dataset listing facilities meeting these criteria^25^ was used to see if these designations applied to any locations in our sample based on the name and location of the facility.

#### Medically Underserved Areas (MUAs)

MUA is a federal designation based on access to primary care services and population characteristics such as infant mortality, poverty level, and percentage of elderly residents.^22^ They can be county-wide or be made up of groups of census tracts within a county. Census tract ID numbers were matched to the MUA dataset for California^25^ to determine MUA status for each primary practice location.

#### The Area Deprivation Index (ADI)

The Area Deprivation Index (ADI) was utilized to assess the level of disadvantage at the primary work location.^26–28^ This measure, independent of shortage area criteria, incorporates various social factors such as income, education, employment, and housing quality to determine disadvantage across census tract groups. Developed by researchers at the University of Wisconsin-Madison, the ADI is available through the Neighborhood Atlas tool,^26^ providing two scores for each location—a state-level score (on a scale of 1–10) and a federal-level score (on a scale of 1–100), with higher scores indicating greater disadvantage. By inputting the fellows’ workplace addresses, their practice locations’ state disparity scores were determined. The ADI scores were then used for criterion validity to statistically assess whether fellows receiving scholarships had higher ADI scores and if representing a community in a federally designated underserved or shortage area correlated with higher ADI scores.

### Analyses

To assess the program’s outreach effectiveness, scholarship availability was tracked annually, calculating the proportion of fellows receiving scholarships. The primary practice locations of fellows were analyzed to determine federal designation status, including HPSA PC, HPSA MH, PCSA, FQHC, and MUA, with a combined HPSA indicator created. An overarching binary variable, “Any Disadvantage Category,” was established, indicating if criteria were met for any federally defined category. ADI scores (1–10) were analyzed for frequency distribution, yielding median scores and proportions of highest (9 or 10) and lowest (1 or 2) scores. Analyses were conducted for the total sample and by cohort year. For criterion validity, Wilcoxon sign-rank tests compared California ADI scores between those with and without scholarships and within the “Any Disadvantage Category.” Chi-square tests examined differences in proportions of fellows serving in areas with the highest ADI scores (9 or 10). ArcMap 10.8.2 was utilized to map TNT fellows at the county level in California based on average state-level ADI per county, visualizing recruitment effectiveness and scope.

## RESULTS

**Figure 1:**
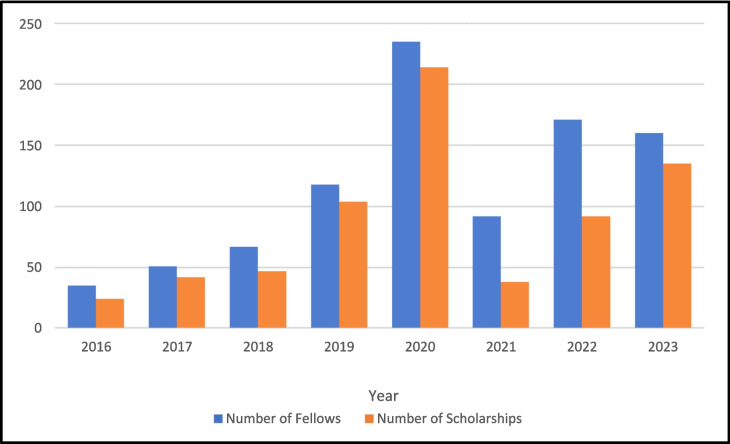
Number of scholarships awarded per year. Bars on the right represent the scholarships awarded while bars on the left represent the total number of fellows for each year.

Since the program’s inception, 929 fellows have completed the TNT fellowship. From 2016 to 2023, 696 (75%) of fellows were awarded scholarships (Table 1). Distribution of scholarships per year is shown in Fig. 1. The majority of scholarships were awarded from HCAI, formerly the Office of Statewide Health Planning and Development (OSHPD). Because primary work location data were only available for years 2021–2023, 423 TNT fellows were included in the practice location analysis. Seventy fellows with primary practice locations outside of CA were excluded. Six CA fellows with workplace addresses that could not be verified were excluded. After thus excluding 76 fellows, the practice location sample comprised 347 fellows practicing in CA at an address that could be matched to at least one of the source databases. A significant proportion of fellows (51.8%, *n* = 171) reported being informed about this fellowship by a colleague or peer, while 16% (*n* = 53) indicated they learned about it directly from TNT alumni (Table 2). Of 347, 88.8% received scholarships. Among the 11.2% not receiving scholarships (*n* = 39), 18 (46%) cared for patients in underserved areas. Summary statistics characterizing the fellows in the analysis sample including demographic characteristics (sex, race/ethnicity), medical specialty, and years since degree are provided in Table 2.

**Table 1:** Scholarships Awarded from 2016 to 2023

Type of scholarship	2016	2017	2018	2019	2020	2021	2022	2023	All years
Alameda Health Consortium	8	11	10	9	4	-	-	-	42
California Inland Empire Health Plan (IEHP)	-	18	10	7	9	12	7	11	74
Partnership	6	6	-	-	-	-	-	-	12
Santa Cruz County	2	2	2	-	-	-	-	-	6
Solano County	5	2	-	-	-	-	-	-	7
UCD Student Health	3	3	-	-	-	-	-	-	6
LA CARE	-	-	10	5	5	3	-	-	23
Cedars Sinai	-	-	-	30	7	16	-	-	46
Fresno County	-	-	15	1	-	-	-	-	16
Redwood Community Health Coalition	-	-	-	8	-	-	-	-	8
UC Davis Health Community Integration	-	-	-	4	3	-	-	-	7
Office of Statewide Health Planning and Development/HCAI	-	-	-	40	186	7	85	124	402
**Total scholarships**	24	42	47	104	214	38	92	135	696
**Number of fellows**	35	51	67	118	235	92	171	160	929
**Percentage of fellows that received scholarships**	68.6%	82.4%	70.1%	88.13%	91.1%	41.3%	53.8%	84.4%	74.9%

**Table 2:** Sample Demographics

**Cohort** (*n* = 347)	**Frequency**	**Percent**
2021	57	16.4%
2022	151	43.5%
2023	139	40.1%
**Sex** (*n* = 347)	**Frequency**	**Percent**
F	253	72.9%
M	91	26.2%
Non-binary	2	0.6%
Not stated	1	0.3%
**Race-ethnicity** (*n* = 324)	**Frequency**	**Percent**
White/Caucasian	99	30.55%
Asian	99	30.55%
Black/African-American	34	10.5%
Hispanic/Latinx	60	18.5%
Native American	3	0.9%
More than one race	22	6.8%
Other	7	2.2%
**Years since degree** (*n* = 341)	**Mean (sd)**	**Range**
	10.43 (9.4)	0–46
**Discipline** (*n* = 347)	**Frequency**	**Percent**
MD	153	44.1%
NP	113	32.6%
PA	44	12.7%
DO	33	9.5%
Other	4	1.1%
**Specialty** (*n* = 347)	**Frequency** (allowed multiple selections)	**Percent**
Family medicine	275	79.3%
Internal medicine	46	13.3%
Pediatrics	21	6.1%
Women’s health (OB/Gyn)	13	3.1%
Other	27	7.8%
**Recruitment Source (*n* = 330)**	**Frequency**	**Percent**
Colleague/peer	171	51.8%
Employer/work	14	4.2%
Online	26	7.9%
TNT alumni	53	16.1%
TNT faculty	8	2.4%
Other (e.g., professional organization, online direct marketing, mailers)	58	17.6%

^*^23 declined to state

**Figure 2:**
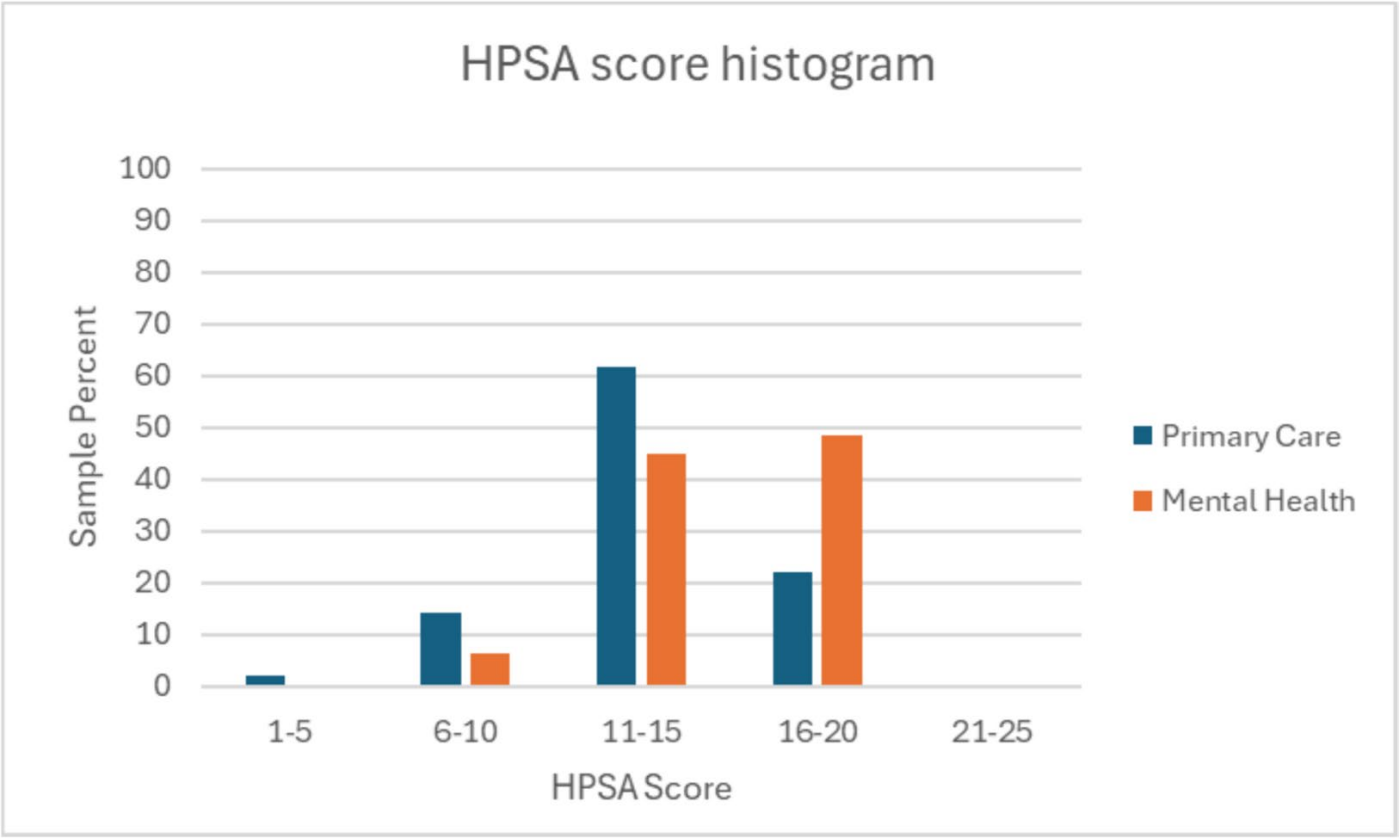
HPSA level score distribution histogram. HPSA scores reflect the level of provider shortage in a given area and are used to prioritize areas for resources and support. The scores range from 0 to 26, with higher scores indicating a greater need for health care services. > 80% of fellows came from a HPSA level > 11.

Of the 347 fellows in the primary practice analysis cohort, 300 (86.5%) served in any disadvantaged category, either HPSA, MUA, or FQHC. HPSA level score histography is included in Fig. 2. Additionally, the proportion of fellows coming from any disadvantaged category has increased from 82.5% in 2021 and 2022 to 89.9% in 2023. The percentages of fellows coming from state and federally qualified shortage areas are summarized in Table 3.

**Table 3:** Shortage Areas and ADI Evaluation of TNT Fellows

Year	Health provider shortage areas (HPSA)				Medically underserved area (MUA) (*n* = 347)	Federally qualified health center (FQHC) (*n* = 347)	Any disadvantage category (*n* = 347)
	Primary care (PCSA)	Primary care provider (HPSA PC)	Mental health provider (HPSA MH)	HPSA shortage area across type (*n* = 343)*			
2021–2023 ( n = 347)	175	146	109	240	131	212	300
	51.0%	42.6%	31.8%	70.0%	37.8%	61.1%	86.5%
2021 ( n = 57)	26	26	19	41	19	33	47
	46.4%	46.4%	33.9%	73.2%	33.3%	57.9%	82.5%
2022 ( n = 151)	68	61	53	99	51	86	128
	45.9%	41.2%	35.8%	66.9%	33.8%	57.0%	84.2%
2023 ( n = 139)	81	59	37	100	61	93	125
	58.3%	42.4%	26.6%	71.9%	43.9%	66.9%	89.9%
**Year**		**Median ADI score**		**% lowest ADI scores (1, 2)**		**% highest ADI scores (9, 10)**	
2021–2023 (*n* = 328)		7		8.8%		32.3%	
2021 (*n* = 55)		7		14.6%		27.3%	
2022 (*n* = 142)		7		10.6%		26.8%	
2023 (*n* = 131)		8		4.6%		40.5%	

^*^4 sites could not be coded in relation to HPSA; 1 in 2021 and 3 in 2022

**Figure 3:**
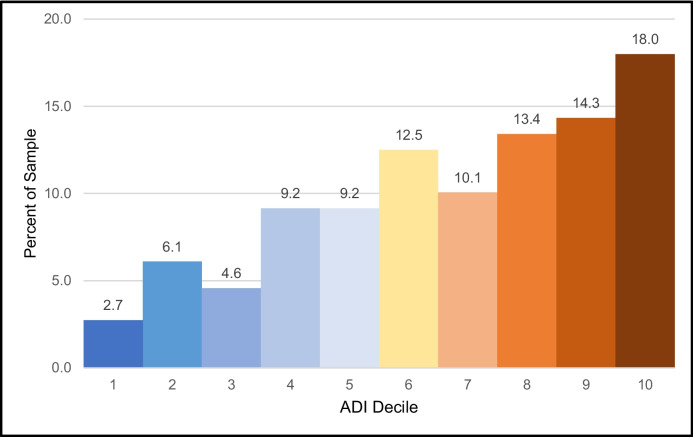
Distribution of disparity scores 2021–2023 (*n* = 328). Area Deprivation Index (ADI) scores distribution for all fellows from 2021 through 2023. An ADI decile of 1 signifies the lowest disparity level whereas an ADI decile of 10 indicates the highest disparity level.

**Figure 4:**
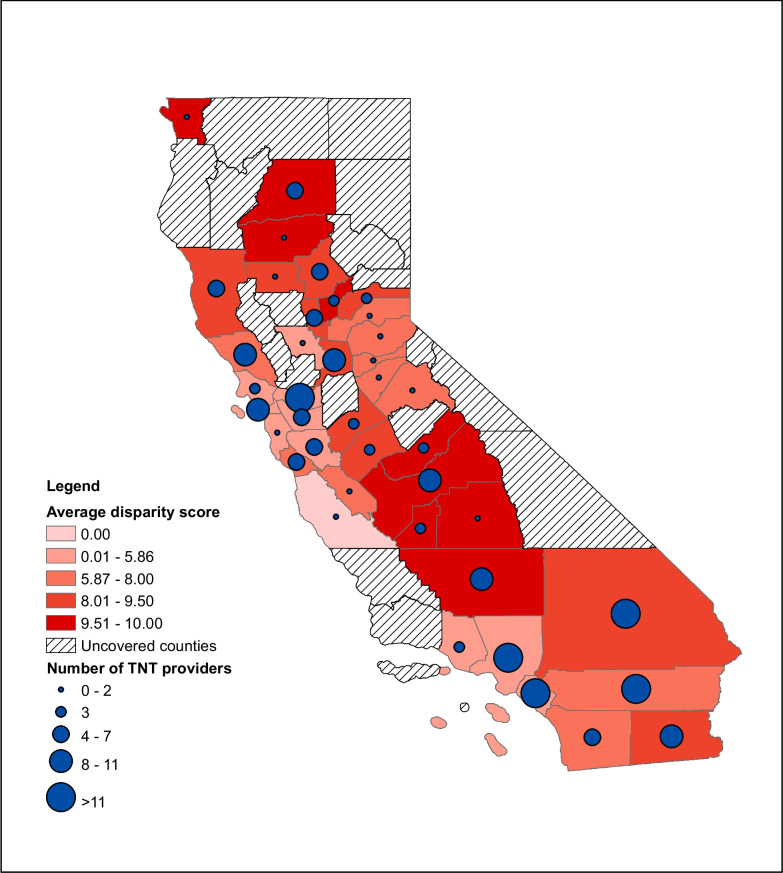
Distribution of Area Deprivation Index (State Level Disparity Score) and total number of TNT providers in 2021–2023. Blue circles indicate the concentration of TNT fellows from each county with smaller circles representing a smaller number of fellows and large circles representing a larger number of fellows. Unshaded, striped regions indicate the counties the program did not reach between 2021 and 2023. Shaded regions specify the average disparity score for the county, with lighter shades corresponding with lower disparity scores and darker shades corresponding with higher disparity scores.

ADI scores were obtained for 328 of the 347 locations in the practice location sample. ADI scores were unable to be determined for 19 addresses at universities or other large hospital institutions as the disparity of the area could not be accurately determined in its census block. From 2021 to 2023, 106 (32.3%) fellows practiced in the highest disadvantaged areas as defined by the ADI. Overall distribution of ADI scores exhibited a negative skew with majority of scores being greater than or equal to 6 (Fig. 3). Figure 4 depicts fellow distribution by county, with ADI averaged per county. When looking at individual cohorts, the 2023 cohort exhibited a shift toward a higher percentage of fellows in the most disparate areas (40.5%) and had a median ADI score of 8 compared to 7 in 2021 and 2022 (Table 3).

Assessment of criterion validity revealed a significant difference in the mean ADI score (*p*_*Wilcoxon*_ < 0.0001) between fellows at practices meeting criteria for Any Disadvantage Category (*Ⴟ* = 7.0) and those that did not (*Ⴟ* = 4.8). Fellows receiving scholarships also exhibited significantly higher (*p*_*Wilcoxon*_ = 0.0002) ADI scores compared to those without scholarships (*Ⴟscholarship* = 6.94, *Ⴟno scholarship* = 4.94). A higher percentage of fellows with scholarships (33.6%) were in practices with ADI scores of 9 or 10 than fellows without scholarships (22%), but the difference was not statistically significant.

## DISCUSSION

This study examines a strategy by TNT to recruit PCPs serving underserved communities in California. Through a comprehensive strategy that included stakeholder-sponsored scholarships, TNT has enrolled the majority of fellows from HPSAs, FQHCs, or MUAs, aligning with its mission to improve access to behavioral healthcare in disadvantaged communities. Since the program’s inception, 929 fellows have completed the TNT fellowship. From 2016 to 2023, 696 (75%) of fellows were awarded scholarships. Practice location data were available for 347 fellows, 88.8% of whom received scholarships. Of the 347 practices, 300 (86.5%) primarily served communities meeting at least one state or federal criterion for medical shortage areas and/or underserved areas. According to ADI scores, 32.3% of practices served areas classified in the highest ADI (ADI decile 9 or 10) with a progressive increase in the proportion of fellows practicing in underserved areas each year; in 2023, 89.9% of practices met federal shortage criteria and 40.5% served areas with the highest deciles of ADI. The high proportion of PCPs practicing in designated shortage areas and the significant percentage of practices with high ADI scores underscore the effectiveness of this recruitment strategy. These results validate this approach to targeting and recruiting PCPs from underserved communities, thereby enhancing access to behavioral healthcare in these areas. The statistically significant increase in the proportion of recruited PCPs practicing in underserved areas (*p* < 0.05) demonstrates the robustness of this recruitment strategy.

The strategy employed by TNT to focus on PCPs from underserved and disadvantaged communities appears to be both sensitive (achieving recruitment of fellows from underserved areas) and specific (with no fellows from adequately served areas receiving scholarships and very few fellows from underserved areas failing to receive a scholarship). Thus, the TNT program’s scholarship criteria are shown to be successful in specifically targeting PCPs practicing in areas of high disparity as determined by their ADI scores and federal and state designations. The fact that over 80% of fellows learned about the program through their employer, peers, or TNT faculty and alumni underscores the success of the local and collaborative recruitment strategy. This suggests that leveraging professional networks, local stakeholders, and community connections significantly enhances program awareness, particularly in disadvantaged areas. Overall, these findings demonstrate the strength of the TNT program’s recruitment strategy in effectively reaching and engaging PCPs serving disadvantaged communities.

One of the key strengths of the TNT program’s strategy is its focus on recruiting *practicing* PCPs from underserved areas. The program’s strategic approach, including partnerships with stakeholders and scholarship support, has contributed to its ability to reach PCPs in high disparity areas with limited access to behavioral health resources. Since its inception, the TNT program has collaborated with stakeholders to provide scholarships to three-fourths of fellows, with numbers growing steadily apart from years 2021 and 2022, likely related to the COVID-19 pandemic.

TNT differs from existing programs and efforts to address the behavioral healthcare gap in primary care. Many programs either target graduate medical education trainees rather than PCPs in active practice^16,18^ and/or focus on integrating behavioral health specialists within the primary care setting^17,29^ instead of equipping PCPs with the skills to treat behavioral health concerns directly and practically within the settings where they see patients. In addition to targeted recruitment, TNT aims to equip PCPs with the necessary skills confidently and accurately to address their patients’ behavioral health concerns. Importantly, TNT was shown to enhance antidepressant prescription behavior in a sample of PCPs from underserved areas in California.^30^ In that study, PCPs from the California Inland Empire Health Plan (IEHP) who completed the TNT fellowship exhibited long-term increases in antidepressant prescription rates compared to IEHP providers who did not complete TNT. IEHP is one of the top ten largest health plans in California’s Medicaid Program (Medi-Cal) that serves a racially/ethnically diverse population (of 1.6 million residents) in Riverside and San Bernardino counties that qualify for Medi-Cal.^31^ The findings from the study suggest that the TNT program increased clinical focus on mental illness and behavioral health concerns, thus improving access to behavioral health services within the primary care setting in one of the areas of greatest need in California.^32^ With the continuation of the program’s recruitment strategy and focus on equipping PCPs with improved knowledge, skills, and attitudes towards providing behavioral healthcare, the TNT program has the potential to reduce disparities and enhance access to quality care in disadvantaged communities.

### Limitations and Considerations

This study design did not include a control program which limits the ability to establish a causal relationship between the program strategy and the observed outcomes. Additionally, the timeframe of the available primary work location data was limited and reliance on self-reported primary work addresses introduces the possibility of inaccuracies or incomplete information, potentially affecting the accuracy of the results. The verification of work locations was conducted solely by the sponsor organizations, and the outcomes of these verifications were not available for this study. Furthermore, while a clinic can be in an area that is considered a shortage area, we are unable to ensure that the residents in the area are representative of the patients seen by the provider since several factors (e.g. transportation, disability, ability to pay, insurance coverage) can affect access to care regardless of its availability.^33^

Future research should focus on assessing the long-term outcomes of the TNT program, including patient outcomes and cost-effectiveness analyses in these areas of increased disparities. Evaluating the sustained impact of the program including knowledge, skills, and attitudes towards behavioral healthcare in PCPs who practice in higher disparity areas can provide valuable insights into methods to combat disparity in medical access. Furthermore, investigating the program’s impact on healthcare utilization patterns and healthcare outcomes in disadvantaged communities would provide a comprehensive understanding of its benefits. Exploring potential strategies to address barriers to participation, such as financial constraints or time commitments, will help ensure broader access to quality behavioral healthcare in disadvantaged communities.

## CONCLUSION

The TNT program was able to effectively recruit fellows practicing in underserved and disadvantaged communities. By enhancing PCP training in behavioral health, the TNT program hopes to reduce the access gaps in behavioral healthcare with the potential to improve the well-being of vulnerable populations in disadvantaged areas and promote health equity. The program’s innovative and targeted approach for recruitment exemplifies its commitment to providing quality care and addressing the critical behavioral health needs of underprivileged populations. Finally, this strategy can be used as a framework to achieve recruitment of physicians who practice in areas of healthcare services shortage and disadvantaged communities.

## Data Availability

The data supporting the findings of this study are available upon reasonable request, pending appropriate regulatory approvals and institutional data-sharing agreements.

## Appendix

See Table 4

**Table 4:** Scholarship Qualification Criteria

Type of scholarship	Qualifications
Alameda Health Consortium	Provider had to work at health center within Alameda Health Consortium network
IEHP	IEHP contracted provider, non-resident physician
Partnership	Providers working for Partnership health plan
Santa Cruz County	Providers working in Santa Cruz County
Solano County	Providers working in Solano County
UCD Student Health	Providers working for UC Davis Student Health
LA CARE	Provider must work in LA Service Planning Area (SPA) 1, 2, 3, and 7
Cedars Sinai	Provider must work in LA Service Planning Area (SPA) 2*, 4, 5, 6, and part of 8
Fresno County	Provider must work in Fresno County
Redwood Community Health Coalition	Must work at FQHC in northern California — 1 scholarship per organization (8 organizations total)
UC Davis Health Community Integration	Must work at Elica Health, One Community Health, Sacramento Native American Health Center, WellSpace Health or CommuniCare in Sacramento, CA
Office of Statewide Health Planning and Development/HCAI	1. Be currently employed or have accepted employment as a primary care or emergency provider (MD, DO, NP, PA) in family medicine, internal medicine, OB/GYN, or pediatrics 2. Be currently employed or have accepted employment at a Federally Qualified Health Center/Lookalike (FQHC) or at a qualifying site in Primary Care Shortage Area (PCSA) or a Health Professional Shortage Area-Primary Care (HPSA-PC) in California 3. Be currently employed or have accepted employment at a practice site with at least 50% of patients from a medically underserved population (Uninsured, Medi-Cal, or beneficiaries of another publicly funded program that serves patients who earn less than 250% of the federal poverty level) 4. Currently serve or plan on serving children and youth 25 years of age or younger^†^

^*^SPA 2 qualification started in 2020

^†^Criteria started in 2022
